# Activation of Secondary Metabolism in Red Soil-Derived Streptomycetes via Co-Culture with Mycolic Acid-Containing Bacteria

**DOI:** 10.3390/microorganisms9112187

**Published:** 2021-10-20

**Authors:** Kairui Wang, Ning Liu, Fei Shang, Jiao Huang, Bingfa Yan, Minghao Liu, Ying Huang

**Affiliations:** 1 State Key Laboratory of Microbial Resources, Institute of Microbiology, Chinese Academy of Sciences, Beijing 100101, China; wangkairui1994@163.com (K.W.); fussliu@126.com (N.L.); huangjiao515665@163.com (J.H.); yanbingfa2014@126.com (B.Y.); 2 College of Life Sciences, University of Chinese Academy of Sciences, Beijing 100049, China; 3 Analytical and Testing Center, Beijing University of Chemical Technology, Beijing 100029, China; shangfei@mail.buct.edu.cn

**Keywords:** *Streptomyces*, co-culture, mycolic acid-containing bacteria (MACB), secondary metabolites (SMs), activation of natural products

## Abstract

Our previous research has demonstrated a promising capacity of streptomycetes isolated from red soils to produce novel secondary metabolites, most of which, however, remain to be explored. Co-culturing with mycolic acid-containing bacteria (MACB) has been used successfully in activating the secondary metabolism in *Streptomyces*. Here, we co-cultured 44 strains of red soil-derived streptomycetes with four MACB of different species in a pairwise manner and analyzed the secondary metabolites. The results revealed that each of the MACB strains induced changes in the metabolite profiles of 35–40 streptomycetes tested, of which 12–14 streptomycetes produced “new” metabolites that were not detected in the pure cultures. Moreover, some of the co-cultures showed additional or enhanced antimicrobial activity compared to the pure cultures, indicating that co-culture may activate the production of bioactive compounds. From the co-culture-induced metabolites, we identified 49 putative new compounds. Taking the co-culture of *Streptomyces* sp. FXJ1.264 and *Mycobacterium* sp. HX09-1 as a case, we further explored the underlying mechanism of co-culture activation and found that it most likely relied on direct physical contact between the two living bacteria. Overall, our results verify co-culture with MACB as an effective approach to discover novel natural products from red soil-derived streptomycetes.

## 1. Introduction

Natural products (NPs), or their semi-synthetic derivatives, are important sources of lead compounds in drug discovery [1,2]. More than 50% of clinically used antibiotics were derived from filamentous Gram-positive bacteria of the genus *Streptomyces* [3,4]. Analysis of the genome sequences of *Streptomyces* and related genera revealed that they may contain a great variety of secondary metabolite (SM) biosynthetic gene clusters (BGCs) encoding novel NPs [5,6]. However, most of these BGCs remain silent under laboratory conditions, thus triggering research in the sense of developing activating strategies for the genome mining of microbial NPs [7,8,9].

Several previous studies reported that microbial cryptic BGCs could be activated by co-culturing the host strains with other species [10,11,12,13], partially due to mimicking the in situ microbial interactions in the original environment where microorganisms coexist [14]. However, traditional co-culture methods require large-scale screening to find ideal microbial combinations, and thus, is laborious and difficult to apply at scale. In recent years, it has been shown that co-cultures with mycolic acid-containing bacteria (MACB) widely activate cryptic SM-BGCs in streptomycetes [15]. Hitherto, around 40 novel NPs have been discovered from *Streptomyces*, and rare actinobacteria by co-culture with MACB strains. These compounds comprise a variety of chemical scaffolds and bioactivities, validating this SM-BGC activation method of co-culture [16,17,18].

Red soils are widely distributed in tropic and subtropical areas of southern China. These soils are acidic, oligotrophic, and rich in iron and aluminum oxides, and thus provide ideal habitats for acidophilic actinobacteria [19]. Our recent studies have shown that red soil-derived streptomycetes are prolific NP producers [19], and have identified several NPs with novel scaffolds or modifications from these strains, as exemplified by azolemycins [20], NC-1 [21], and mycemycins [22]. Meanwhile, the in silico genome-mining of red soil-derived streptomycetes also reveals that these strains contain numerous unidentified SM-BGCs, the products of which remain to be unraveled.

To activate the silent SM-BGCs of red soil-derived streptomycetes for NPs discovery, we selected 44 bioactive *Streptomyces* isolates and co-cultured them with four MACB of different species in a pairwise manner. Metabolites of the co-cultures were subjected to multi-spectroscopic analyses and bioactivity assay, which showed that the MACB strains effectively activated secondary metabolism in most of the streptomycetes. We also tried to explore the underlying mechanism of co-culture activation. Results of the study gain a deep insight into the NP biosynthetic potential of red soil-derived streptomycetes.

## 2. Materials and Methods

### 2.1. Strains and Media

The strains used in this work are listed in Table S1. Eight MACB were isolated from soils collected in Haixi Mongolian and Tibetan Autonomous Prefecture, China [23], and two MACB and all *Streptomyces* strains were from red soils collected in Jiangxi Province, China [19,24]. All these strains were preliminarily identified by 16S rRNA gene sequencing in our previous studies [19,23,24]. Three indicator strains from different phyla were used for antimicrobial activity assay: *Micrococcus luteus* CGMCC 1.2567 (Gram-positive bacterium) and *Trichoderma viride* CGMCC 3.1913 (fungus) were obtained from the China General Microbiological Culture Collection Center (CGMCC), and extended-spectrum β-lactamase (ESBL)-producing *Escherichia coli* 4-1 (Gram-negative bacterium) was obtained from the Weifang Medical University, Shandong Province, China.

A YGGS medium (glucose 5.0 g, soluble starch 20.0 g, glycerin 20.0 g, yeast extract 3.0 g in 1 L dd-H_2_O, pH 7.2) [25] was used for the co-culture and pure culture of strains. A TSB medium (pancreatic digest of casein 17.0 g, papaic digest of soybean 3.0 g, dextrose 2.5 g, NaCl 5.0 g, K_2_HPO_4_ 2.5 g in 1 L dd-H_2_O, pH 7.1–7.5) was used for seed culture. A GYM agar (yeast extract 4.0 g, malt extract 10.0 g, glucose 4.0 g, CaCO_3_ 2.0 g, agar 15.0 g in 1 L dd-H_2_O) was used for recovering strains from glycerol stocks. An LB agar (tryptone 10.0 g, yeast extract 5.0 g, NaCl 10.0 g, agar 15.0 g in 1 L dd-H_2_O) and PDA (glucose 20.0 g, potato powder 6.0 g, agar 15.0 g in 1 L dd-H_2_O) were used to culture the indicator bacteria and fungus, respectively.

### 2.2. Co-Culture and Pure Culture of Strains

After the incubation of the strains on a GYM plate for 2–3 days, an agar block of about 1 cm^2^ with bacterial lawn was cut out and transferred into a 250 mL shake flask containing 50 mL of TSB medium for seed culture. The seeds of *Streptomyces* and MACB were cultured at 28 °C on a rotary shaker at 160 rpm for 3 and 2 days, respectively. Then, 3 mL of *Streptomyces* and 1 mL of MACB seed cultures were co-transferred into a 250 mL flask containing 100 mL of YGGS medium and fermented at 28 °C, 220 rpm for 7 days. Pure culture controls were performed similarly but with single strains.

Each experiment was repeated in triplicate in this study.

### 2.3. SM Extraction, Isolation, and Analysis

The resulting cultures were collected and extracted three times with an equal volume of ethyl acetate. The extracts were combined and concentrated in vacuo to evaporate the solvent, and the residue was re-dissolved in 1 mL of methanol. An HPLC analysis was carried out with a Shimadzu SPD-M20A HPLC system, using a Waters Xbridge ODS column (4.6 × 150 mm, 5 μm) with a linear gradient of MeOH/H_2_O (see Table S2). The injection volume of the sample was 20 μL. The Dionex 3000 RS system was used to set the temperature at 30 °C and the flow rate at 1.0 mL/min; the elution curves of metabolites were monitored at 220, 254, and 300 nm, respectively. Differences in the secondary metabolism between the co-cultures and pure cultures were determined by comparing their HPLC profiles based on the retention time and UV absorption spectra of peaks. Metabolites corresponding to the differential HPLC peaks were then collected and subjected to UHPLC-HRMS (Waters Xevo G2 quadrupole time of flight-ultra performance liquid chromatography, and mass spectra scanning from 100 to 2000 atomic mass units) to obtain their accurate molecular weights (MWs). The resultant mass spectrum data were analyzed by the Mass Lynx v 4.1 software system.

Compounds were identified by the comparison of MWs, UV spectra, and retention times with published chemical data from standard databases (Dictionary of Natural Products [DNP] on DVD, version 22.2 and on web, version 30.1 [http://dnp.chemnetbase.com/, accessed on 1 September 2021]; ChemSpider [http://www.chemspider.com/, accessed on 2 September 2021]) and references. The activated metabolites with characteristic information unmatched with that in the databases were inferred as putative new products. Some of these compounds were subjected to nuclear magnetic resonance (NMR) spectroscopic analysis (Bruker AVIII 500 MHz NMR spectrometer, Bruker, Karlsruhe, Germany) for further structure elucidation.

### 2.4. Bioactivity Assay

Bioactivities of the fermentation extracts were tested against *M*. *luteus*, ESBL-producing *E*. *coli*, and *T*. *viride* using agar-well diffusion assay. Twenty μL of each extract were added into a punched hole (7 mm in diameter) in LB/PDA plates containing indicator strains. The plates were then cultured at 37 °C for 12 h for bacterial indicators or at 28 °C for 48 h for the fungus. Antimicrobial activity was estimated by measuring the diameter of the inhibition zones: positive (7 mm < diameter ≤ 9 mm) and strongly positive (diameter > 9 mm).

### 2.5. Non-Contact Co-Culture of Streptomyces sp. FXJ1.264 and Mycobacterium sp. HX09-1

The co-culture was carried out in a device of two connected culture compartments separated by a 0.22-μm polyether sulfone (PES) membrane (Figure S1). Each of the compartments contained 50 mL YGSS medium and were inoculated with 3 mL seed culture of *S*. sp. FXJ1.264 or 1 mL seed culture of *M*. sp. HX09-1. The device only allowed substance exchange between the compartments, but the cells of the two strains could not contact each other. For the control groups, only one compartment in the device was inoculated, with either a single strain or two strains mixed. The device was fixed on a shaker and the strains were fermented at 28 °C, 220 rpm for 7 days. The fermentation metabolites from each compartment were analyzed using the method described in Section 2.3.

### 2.6. Co-Culture of S. sp. FXJ1.264 and Heat-killed M. sp. HX09-1

*M*. sp. HX09-1 was cultured in 250 mL flasks each containing 50 mL of the YGGS medium for 2 or 7 days. The culture broths were then heated at 121 °C for 20 min to kill the cells. After cooling down to room temperature, the flask was added with 50 mL fresh YGGS medium and 3 mL seed culture of *S*. sp. FXJ1.264, and the resulting culture was incubated at 28 °C, 220 rpm for 7 days. The fermentation metabolites were analyzed as above.

## 3. Results

### 3.1. Preliminary Evaluation of the Activation Ability of MACB and Selection of Red Soil-Derived Streptomycetes

**Figure 1:**
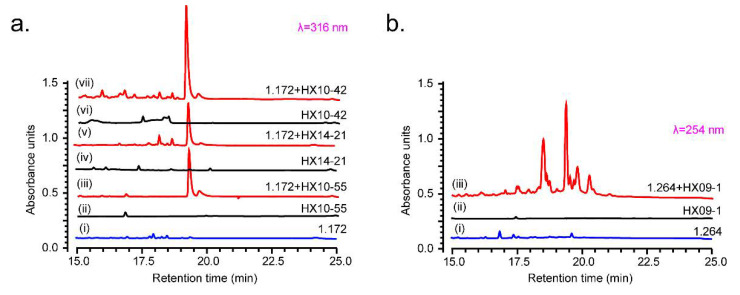
Preliminary evaluation of the activation ability of mycolic acid-containing bacteria (MACB). (**a**) HPLC analysis of the fermentation extracts of *S*. sp. FXJ1.172 co-cultured with different MACB and the extracts of their pure cultures; (**b**) HPLC analysis of the fermentation extracts of *S*. sp. FXJ1.264, *M*. sp. HX09-1, and their combined culture.

Ten MACB candidates (listed in Table S1) and two known NPs producer strains that were isolated from red soil, *Streptomyces* spp. FXJ1.172 and FXJ1.264 [19], were used for preliminary co-culture evaluation. The HPLC profiles of metabolites showed that three of the MACB (*Mycobacterium* sp. HX10-42, *Nocardia* sp. HX14-21, and *Rhodococcus* sp. HX10-55) obviously activated *S.* sp. FXJ1.172 to produce new peaks compared to their individual pure cultures (Figure 1a). Meanwhile, a series of unique peaks were detected in the combined culture of *S.* sp. FXJ1.264 and *Mycobacterium* sp. HX09-1 (Figure 1b). The other six MACB did not exhibit activation ability when co-cultured with the two *Streptomyces* strains. Therefore, the above four strains of MACB were chosen for subsequent co-culture. In addition, based on the antimicrobial activity and 16S rRNA gene similarity of the red soil-derived *Streptomyces* strains [19,24], 44 bioactive streptomycetes with abundant diversity were preferentially selected for this study (Table S1). The selected MACB and streptomycetes thus formed 176 (4 × 44) co-culture pairs.

### 3.2. Co-Culture with MACB Changed the SM Profiles of Streptomycetes

**Figure 2:**
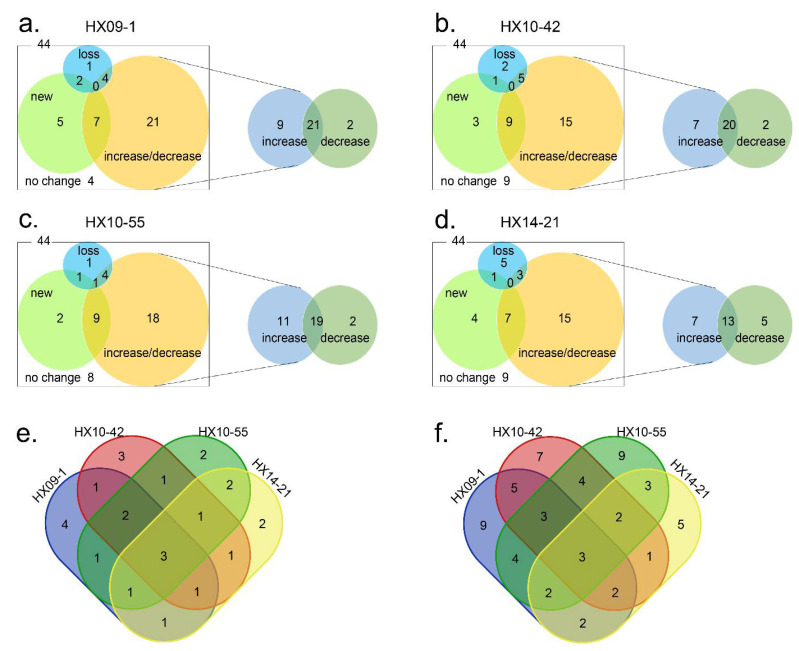
Venn diagrams showing effects of co-culture with four different MACB on the SM profiles of 44 red soil-derived streptomycetes. Pure cultures were used as controls. The numbers represent the number of streptomycetes or co-culture pairs. (**a**–**d**) Summarization of the effects of co-culture for each MACB. Each HPLC profile was classified according to the aforementioned categories: increase/decrease, the yields of original metabolites produced in pure cultures increased/decreased in the co-cultures (the integral area of HPLC peaks changed by more than 30%); new, “new” metabolites with unique retention time and/or UV absorption that exclusively appeared in the co-cultures; loss, original pure culture metabolites lost in the co-cultures; and no change, no difference in SM profiles was detected between the co-cultures and pure cultures. The increase/decrease part is subclassified into increase, decrease, and both increase and decrease in the right panel. (**e**) Overlaps of the effects between MACB on inducing “new” metabolites. (**f**) Overlaps of the effects between MACB on increasing the yields of original metabolites.

HPLC analysis showed changes in SM profiles of most (82.9%, 146/176) of the co-cultures compared to the pure culture counterparts (Figure 2 and Table 1). The differences of metabolites were characterized by four patterns of HPLC peaks: the increase/decrease in metabolite production (the integral area of peaks changed by more than 30%), appearance of new metabolite peaks, loss of original peaks, and no change. The comparison results were mostly a combination of the above patterns due to the complex secondary metabolism in Streptomyces (Figure 2). For example, compared to the pure cultures, co-culture with M. sp. HX09-1 changed the secondary metabolism of 40 Streptomyces strains. Among them, 30 and 23 strains enhanced and decreased the production of some original metabolites, respectively, 14 strains produced new metabolites that were not found in the pure cultures, and seven strains lost some metabolites (Figure 2a). M. sp. HX10-42, R. sp. HX10-55, and N. sp. HX14-21 exhibited similar activation ability (Figure 2b–d) (Table 1). In summary, 29.5% (52/176) of the co-cultures activated the production of “new” SMs and 60.8% (107/176) of the co-cultures increased the production of original metabolites (Table 1). For all four MACB strains, the proportion of positive impact (increase in production and/or appearance of “new” metabolites) on the secondary metabolism of Streptomyces is significantly higher (p < 0.01) than that of negative impact (decrease in production and/or loss of metabolites), as shown in Table 1. Only four of the co-cultured streptomycetes were not activated by any of the MACB. These results indicated that the four MACB strains effectively induced NPs biosynthesis or enhanced their yields in the co-cultured Streptomyces strains.

**Figure 3:**
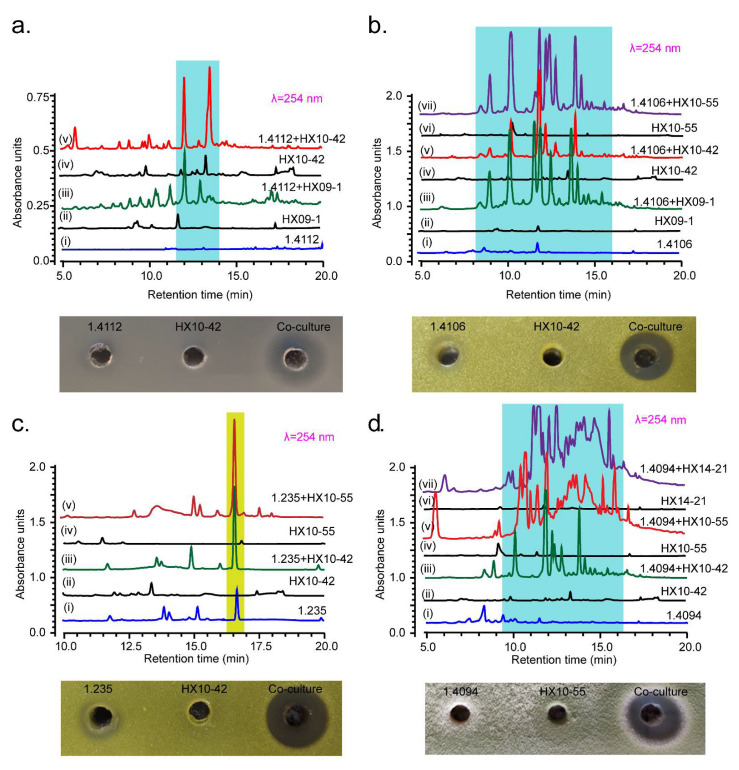
HPLC analysis of changes in SM spectra and agar-well diffusion test of emerging antimicrobial activity in the co-cultures compared to the pure cultures. (**a**) *S*. sp. FXJ1.4112 co-cultured with MACB produced “new” metabolites (blue shadowed) and strong antibacterial activity against *E*. *coli*; (**b**) *S*. sp. FXJ1.4106 co-cultured with MACB produced a series of “new” metabolites (blue shadowed) and strong antibacterial activity against *M*. *luteus*; (**c**) *S*. sp. FXJ1.235 co-cultured with MACB produced a higher yield of the original metabolite (yellow shadowed; the integral area of HPLC peaks was over twice that of control) and strong antibacterial activity against *M*. *luteus*; (**d**) FXJ1.4094 co-cultured with MACB produced a series of “new” metabolites (blue shadowed) and strong antifungal activity against *T*. *viride*. Antimicrobial activity was estimated by measuring the diameter of the inhibition zones: positive (7 mm [hole diameter] < diameter ≤ 9 mm) and strongly positive (diameter > 9 mm).

We noticed some overlaps in the activation effects (Figure 2e,f). That is, the same *Streptomyces* strain could be activated by different MACB to produce similar new HPLC peaks, or to increase the yields of similar original metabolites, as exemplified by *Streptomyces* spp. FXJ1.4112, FXJ1.4106, FXJ1.235, and FXJ1.4094 (Figure 3a–d). This result suggests that *Streptomyces* might use some general weapons when facing different MACB, and that the shared mycolic acids present in different MACB might be one of the key substances to change the secondary metabolism of *Streptomyces*.

### 3.3. Changes of Antimicrobial Activity in the Co-Cultures

Besides analyzing the SM profiles (HPLC detection), we also used agar-well diffusion assay to monitor the difference of antimicrobial activity between the co-culture and pure culture extracts. Among the 176 co-culture pairs, 21 pairs exhibited distinguishing antimicrobial activities compared to the pure culture controls (Table 2), with 15 pairs displaying new activities and the other 6 pairs showing improved activities. Intriguingly, no decrease or loss of pre-existing antimicrobial activities was observed in the co-cultures.

The 21 co-culture pairs mentioned above all showed the “Increase/New” patterns of SM changes based on their HPLC profiling (Table 1). Thus, we speculated that the additional bioactivities might be attributed to the induced or increased production of metabolites in the combined cultures. Indeed, changes of antimicrobial activity in some co-cultures were correlated to the detected alterations of SM spectra. For instance, the co-culture of *S.* sp. FXJ1.4112 and *M.* sp. HX10-42 displayed antibacterial activity against *E. coli*, which was not observed in the pure cultures (Figure 3a and Table 2); correspondingly, two novel product peaks were detected in the HPLC profile of co-culture extracts (Figure 3a). After isolation and purification, the metabolites corresponding to the two peaks (further deduced as collinomycin analogs) showed antibacterial activity against *E. coli*. Similar results were also observed in the co-cultures of *S.* sp. FXJ1.4106 and MACB (Figure 3b and Table 2). In addition, co-culture also improved antibacterial activity of some streptomycetes. For example, the pure culture of *S.* sp. FXJ1.235 had only weak antibacterial activity against *M*. *luteus*, while the co-cultures with *M.* sp. HX10-42 and *R.* sp. HX10-55 had obvious antibacterial activity against *M*. *luteus* (Figure 3c and Table 2). The striking increase (2.08 and 2.70 times) in the yield (integral area of HPLC peaks) of the original antibacterial product in the co-cultures might contribute to the resultant enhanced activity (Figure 3c). These results confirm that co-culture can stimulate *Streptomyces* to produce bioactive NPs. However, not all the changes of activity in the co-cultures could be correlated to the changes of HPLC profiles. The co-culture of *S.* sp. FXJ1.4094 and *N*. sp. HX10-55 exhibited obvious antifungal activity against *T. viride*, which was contrary to the result of their pure cultures (Figure 3d and Table 2). But the product components corresponding to the new peaks in the HPLC profile of co-culture extracts (Figure 3d) showed no antifungal activity. Similar results were also observed in co-culturing *Streptomyces* spp. FXJ1.4014, FXJ1.4034, and FXJ1.4037 with MACB. These examples highlight the importance of testing antimicrobial activity as a complementary approach to secondary metabolism comparison, as metabolite differences may be overlooked if the differential compounds display low or no UV absorption in HPLC profiling.

### 3.4. Co-Culture of Streptomyces and MACB Is a Reliable Source of New Compounds

After UHPLC-HRMS analysis of the “new” metabolites appeared in the co-cultures, a total of 49 compounds were identified as putative novel NPs based on their unique MWs and/or UV spectra compared with those in databases and references (Table 3). Among them, 33 (67.3%) compounds were considered as NPs with new skeletons (structural class unknown) because neither their MWs nor UV spectra matched any NPs recorded in the databases or references.

Six putative new-skeleton NPs were detected in the co-culture of *S*. sp. FXJ1.264 and *M*. sp. HX09-1 (Table 3). They were analogues featuring similar UV absorption characteristics (UV: 195, 251, 285, 376 nm), color, and solubility, and were named 1.264HX-**1**–**6** (Figure S2a,b). HRMS results revealed that the MWs of 1.264HX-**1**, 1.264HX-**3**, 1.264HX-**4**, 1.264HX-**5**, and 1.264HX-**6** were 372, 386, 744, 744, and 744, respectively (Table S3 and Figure S2c,d). The MW of 1.264HX-**3** was 14 Da larger than that of 1.264HX-**1**, indicating that it might be a methylated derivative of 1.264 HX-**1** (Figure S2c,d). The MW of 1.264HX-**4** was twice that of 1.264HX-**1**, inferring that it might be a dimer of 1.264HX-**1**, and so do 1.264HX-**5** and 1.264HX-**6** (Figure S2c,d). The MW of compound 1.264HX-**2** was not determined owing to a lack of MS ion current in both ESI- and APCI- ion sources.

We isolated and purified compound 1.264HX-**3** from the extracts, which unfortunately, was unstable. HRMS analysis of 1.264HX-**3** supported the molecular formula of C_25_H_22_O_4_, indicating fifteen degrees of unsaturation. ^1^H-NMR analysis of 1.264HX-**3** showed that no matter which peak was set as the standard, there were still many peaks whose integral areas were non-integral. For example, the integral areas of peaks at δ 5.79 and 7.90 ppm were 0.52 and 0.57, respectively, which were far smaller than 1 (presumed to be CH); and peaks at δ 1.18 and 3.80 ppm displayed integral areas of 4.98 and 4.72, respectively, which were much larger than 3 (presumed to be CH_3_). Accordingly, in the ^13^C-NMR spectrum, two or three similar signal bars were seen at each ^13^C chemical shift, which greatly increased the difficulty of spectral analysis (Table S4). Therefore, the possible carbon atoms and their directly connected hydrogen atoms of 1.264HX-**3** were tentatively numbered by analyzing the ^1^H, ^13^C, and HSQC spectra, as shown in Table S4. Partial structural fragments of this compound were elucidated based on the current NMR data (Figures S3–S10), which revealed that it belongs to polyketides.

Meanwhile, some putative new analogues of known compounds were identified from the co-cultures as well. The co-culture of *S.* sp. FXJ1.4099 and *M.* sp. HX10-42 activated the production of fogacin and three putative new analogues with similar HPLC retention times and UV absorptions [26,27]. The co-culture of *S.* sp. FXJ1.4106 and *M.* sp. HX09-1 led to the production of a new compound bearing highly similar UV absorption, but different MW to tetracycline [28]. Two compounds with unpublished MWs produced by the co-culture of *S.* sp. FXJ1.4112 and *M.* sp. HX10-42 were deduced as collinomycin analogs due to their dark red color and characteristic UV absorption [29,30]. We speculated that the co-culture of *S.* sp. FXJ1.4111 and *R.* sp. HX10-55 produced actinoperylone and three putative new analogues, which had a similar UV absorption [31]. In addition, a putative new iron siderophore was discovered from the co-cultural broth of *S.* sp. FXJ1.4102 and *N.* sp. HX14-21, on account of its characteristic UV absorption at 404 nm and the rapid emergence of red color in solution after the addition of ferric ion [19]. Together, these data confirm that the co-culture of *Streptomyces* and MACB is a reliable source of novel NPs.

### 3.5. Direct Physical Contact between Streptomycetes and MACB Is Essential to Induce SMs in the Co-Culture

**Figure 4:**
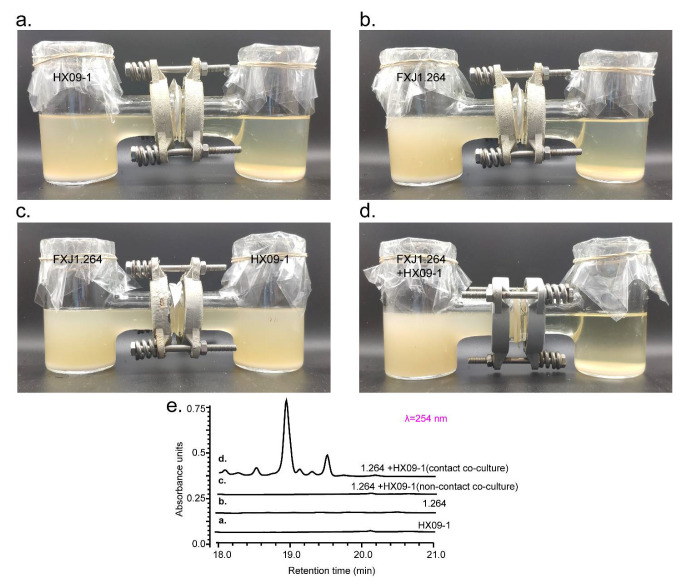
Contact and non-contact co-cultures of *S*. sp. FXJ1.264 and *M*. sp. HX09-1 and related HPLC profiling. (**a**) *S*. sp. FXJ1.264 was only inoculated in the left compartment; (**b**) *M*. sp. HX09-1 was only inoculated in the left compartment; (**c**) *S*. sp. FXJ1.264 and *M*. sp. HX09-1 were individually inoculated in the left and right compartment, respectively; (**d**) *S*. sp. FXJ1.264 and *M*. sp. HX09-1 were inoculated in the left compartment; (**e**) HPLC analysis of fermentation extracts from (**a**–**d**).

We took the co-culture of *S*. sp. FXJ1.264 and *M*. sp. HX09-1 as a case to further explore the underlying mechanism of induction. A non-contact device described in method *2.5* was used to investigate whether cell-to-cell contact was required for the activation of SMs. After fermentation for 7 days, samples from the compartments of the device (Figure 4a–d) were plated on fresh YGSS agar and incubated for 5 days. No growth of bacteria was observed for samples from the un-inoculated compartments, validating that the cells of the two strains could not pass through the 0.22 μm filter membrane. HPLC analysis of the fermentation extracts showed that, compared to the pure culture controls (Figure 4a, b), the non-contact co-culture (Figure 4c) produced no new metabolites while the contact co-culture (Figure 4d) still produced a series of new peaks (Figure 4e). Moreover, co-culturing *S*. sp. FXJ1.264 with heat-killed 2- or 7-day-old *M*. sp. HX09-1 cultures did not activate any new metabolites either (Figure S11). These results indicate that the induced production of new metabolites by co-culture of *S*. sp. FXJ1.264 and *M*. sp. HX09-1 probably relies on direct physical contact between the living cells of the two strains, instead of on the molecular elicitors secreted by MACB.

## 4. Discussion

Though MACB have been proved to widely activate cryptic SM-BGCs in actinobacteria [16], it seems that the selection of actinobacteria also plays a non-negligible role in the efficiency of induction. Overall, the effect of MACB activation on *Streptomyces* is significantly better than that on marine *Micromonosporaceae* reported [32] (Figure S12), possibly because *Streptomyces* have a stronger ability to synthesize NPs than *Micromonosporaceae*. In addition, the co-cultures of MACB and red soil-derived *Streptomyces* display a significantly higher ratio of an increase in NP yields than the co-cultures of MACB and *Streptomyces* from other habitats [15] (Figure S12). This difference can be partially attributed to the outstanding biosynthetic capacity and phylogenetic diversity of the streptomycetes we used. These comparisons highlight the importance of strain selection for rational optimization of co-culture in future work.

The novel NPs obtained by the co-culture of *Streptomyces* and MACB prompted us to trace the original producers of these compounds. In silico genome mining of several *Streptomyces* strains and the type strains most closely related to the MACB used in this study reveals that the predicted diversity and number of SM-BGCs in *Streptomyces* (over 40 BGCs per genome) are overwhelmingly higher than those in MACB (less than 25 BGCs per genome) [20,21]. Therefore, it seems plausible that the unique metabolites emerging in the co-cultures may be derived from *Streptomyces*. This speculation is supported by following experimental evidences. (i) The co-culture of the same *Streptomyces* strain with different MACB leads to rediscovery of the same induced metabolites (Figure 1a and Figure 3a–d). (ii) Some of the activated products in co-cultures were known compounds isolated from *Streptomyces*. For example, the co-culture of *S*. sp. FXJ1.4012 and *N*. sp. HX14-21 produced multiple new HPLC peaks, three of which were identified as enterocin, deoxyenterocin, and oligomycin D (Figure S13). Enterocin and deoxyenterocin have been isolated from *Streptomyces*, but not from MACB [33,34], while oligomycin D is a macrolide antibiotic originally discovered from *Streptomyces* [35]. (iii) The induced NPs in the co-culture of *S*. sp. FXJ1.264 and *M.* sp. HX09-1 was also observed in the pure culture of a genetically manipulated mutant of *S*. sp. FXJ1.264 (data not shown). Based on the above bioinformatical and experimental evidences, we conclude that the new metabolites produced by co-cultures were mainly from *Streptomyces*.

Although several studies have reported that co-culture with MACB can change the SM profiles of *Streptomyces* [36,37,38], the specific mechanism of this phenomenon is still unclear. At present, the possible mechanisms mainly include three types: (i) direct physical contact of co-cultured strains [15,25], (ii) the induction role of molecular compounds secreted by MACB [39], and (iii) horizontal gene transfer between strains [40]. In our study, the result of the non-contact co-culture of *S*. sp. FXJ1.264 and *M.* sp. HX09-1 suggests that the production of new metabolites of *S.* sp. FXJ1.264 may not be mediated by molecule exchange between the two strains, but by direct contact between the cells. In order to further test the possibility of horizontal gene transfer during direct contact, we re-isolated the two strains from the co-cultured broth and cultured them separately. We did not find any new compounds from the pure cultures of the two isolated strains, and thus, essentially ruled out the possibility of activating new products by horizontal gene transfer. Moreover, a recent study reported that some SMs of *Streptomyces* could be activated by both living producer strains and heat-killed strains. [41,42]. However, the heat inactivated *M.* sp. HX09-1 strain failed to activate the production of new metabolites in *S*. sp. FXJ1.264 (Figure S11), indicating that the activation effect was not induced by dead cells. To summarize, direct contact between living *M.* sp. HX09-1 and *S*. sp. FXJ1.264 cells is needed to activate the new metabolites from *S.* sp. FXJ1.264. Nevertheless, it is limited in our work to study the activation mechanism using only one pair of co-culture, and other mechanisms may also exist in other co-culture combinations that activate new metabolites. Extensive comparative “-omics” work needs to be done to predict the activating factors in future investigation.

## 5. Conclusions

To explore the cryptic novel NPs encoded by red soil-derived *Streptomyces*, we co-cultured them with MACB and compared the SM spectra and antimicrobial activity of the co-cultures and pure cultures. We found that 82.9% (146/176) and 60.8% (107/176) of the co-culture pairs changed the SM spectra and enhanced the yield of original SMs of the pure cultures, respectively. Moreover, 29.5% (52/176) of the co-culture pairs produced unique SMs not detected in the pure cultures, of which 49 compounds were putative novel NPs. In addition, the bioactivity assay revealed that co-culture could induce and/or enhance the production of bioactive SMs. These results confirm that MACB can effectively alter the secondary metabolism of *Streptomyces*. Furthermore, the activation of new SMs in the co-culture of *S*. sp. FXJ1.264 and *M*. sp. HX09-1 depends on the direct contact of their living cells. In conclusion, the co-culture of MACB and red soil-derived streptomycetes is an efficient and applicable approach for novel NP discovery.

## Figures and Tables

**Table 1:** Proportions of different change patterns in SM profiles of the co-cultures compared to the pure culture counterparts. The numbers represent the number of co-culture pairs.

Pattern(s)	*M*. sp. HX09-1	*M*. sp. HX10-42	*R*. sp. HX10-55	*N*. sp. HX14-21	Total
Increase	30/44	27/44	30/44	20/44	107/176
New	14/44	13/44	13/44	12/44	52/176
Increase/New	39/44	31/44	33/44	25/44	128/176
Decrease	23/44	22/44	21/44	18/44	84/176
Loss	7/44	8/44	7/44	9/44	31/176
Decrease/Loss	26/44	26/44	25/44	22/44	99/176
Change	40/44	35/44	36/44	35/44	146/176

**Table 2:** Differences of antimicrobial activity between the pure cultures and co-cultures.

Streptomycete	Mycolic Acid-Containing Bacteria				Antimicrobial Activity		
	*M*. sp. HX09-1	*M*. sp. HX10-42	*R*. sp. HX10-55	*N*. sp. HX14-21	EC	ML	TV
FXJ1.235							
		+					
			+				
FXJ1.4012							
				+			
FXJ1.4014							
	+						
FXJ1.4034							
	+						
			+				
FXJ1.4037							
	+						
FXJ1.4094							
		+					
			+				
				+			
FXJ1.4104							
	+						
FXJ1.4106							
	+						
		+					
			+				
FXJ1.4112							
	+						
		+					
FXJ1.535							
	+						
		+					
FXJ1.907							
		+					
			+				
				+			

Note: “+”, co-culture with mycolic acid-containing bacteria; 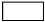, negative; 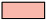, positive; 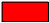, strongly positive; EC, multi-drug resistant *Escherichia coli* 4-1; ML, *Micrococcus luteus* CGMCC 1.2561; TV, *Trichoderma viride* CGMCC 3.1913. Antimicrobial activity was estimated by measuring the diameter of the inhibition zones: positive (7 mm [hole diameter] < diameter ≤ 9 mm) and strongly positive (diameter > 9 mm).

**Table 3:** Putative novel products from co-cultures of the streptomycetes and MACB.

Co-Culture	Compound(s) Induced	Structural Class	Chemical Data	
			UV (MeOH)	MS (*m*/*z*)
FXJ1.172 + HX14-21	2 putative novel compounds	Unknown	221	[M + H] + 603.3187
		Unknown	221	[M + H] + 605.2964
FXJ1.235 + HX10-42	2 putative novel compounds	Unknown	244, 256, 265, 294, 307, 340	[M + H] + 304.0403
FXJ1.235 + HX10-55		Unknown	244, 256, 265, 294, 307, 340	[M + H] + 445.0760
FXJ1.264 + HX09-1	6 putative novel compounds	Polyketide	194, 251, 285, 376	[M − H] − 371.1292
		Polyketide	194, 251, 285, 376	MS no ion current
		Polyketide	194, 251, 285, 376	[M − H] − 385.1433
		Polyketide	194, 251, 285, 376	[M − H] − 743.2671 (dimer of 371.1292)
		Polyketide	194, 251, 285, 376	[M − H] − 743.2645 (dimer of 371.1292)
		Polyketide	194, 251, 285, 376	[M − H] − 743.2634 (dimer of 371.1292)
FXJ1.4038 + HX09-1	1 putative novel compound	Unknown	201, 249, 336	[M + H] + 347.0921
FXJ1.4059 + HX10-42	1 putative novel compound	Unknown	199, 219, 276	[M + H] + 319.0830
FXJ1.4064 + HX10-55	3 putative novel compounds	Unknown	205, 289	[M + H] + 477.3209
		Unknown	205, 294	[M + H] + 589.3013
		Unknown	218, 275	[M + H] + 617.3292
FXJ1.4075 + HX10-55	2 putative novel compounds	Unknown	248, 343	[M + H] + 253.1188
		Unknown	221, 311, 351	[M + H] + 569.0939
FXJ1.4087 + HX10-55	1 putative novel compound	Unknown	197, 224, 272, 336	[M + H] + 288.1239
FXJ1.4094 + HX09-1	2 putative novel compounds	Unknown	276, 373	[M + H] + 509.2658
		Unknown	None	[M − H] − 525.2604
FXJ1.4097 + HX09-1	2 putative novel compounds	Unknown	223, 265	[M + H] + 639.3022
		Unknown	225, 267	[M + H] + 671.3289
FXJ1.4097 + HX10-42	3 putative novel compounds	Unknown	197, 245	[M + H] + 299.1128
		Unknown	197, 245	[M + H] + 597.2175 (dimer of 299.1128)
		Unknown	224, 266	[M + H] + 954.4636
FXJ1.4099 + HX10-42	3 putative novel compounds related to fogacin	Polyketide	221, 274, 340	[M + H] + 863.2872
		Polyketide	221, 277	[M + H] + 893.2611
		Polyketide	221, 271, 335	[M + H] + 909.2637
FXJ1.4099 + HX10-55	4 putative novel compounds	Unknown	234, 276, 340	[M + H] + 317.2076
		Unknown	228, 275, 340	[M + H] + 453.1749
		Unknown	220, 275, 310	[M + H] + 457.1614
		Unknown	229, 275, 330, 350	[M + H] + 607.1823
FXJ1.4102 + HX10-55	1 putative novel compound	Unknown	199, 286	[M + H] + 365.1027
FXJ1.4102 + HX14-21	2 putative novel compounds	Unknown	237, 318, 332	[M + H] + 622.3491
		Siderophore	195, 225, 274, 404	[M + H] + 654.2662
FXJ1.4106 + HX09-1	1 putative novel compound related to tetrodecamycin	Polyketide	206, 254	[M + H] + 335.1494
FXJ1.4106 + HX10-42	2 putative novel compounds	Unknown	194, 240	[M + H]- 229.0674
		Unknown	None	[M + H] + 782.5692
FXJ1.4110 + HX14-21	1 putative novel compound	Unknown	227, 272, 338	[M + H] + 261.1125
FXJ1.4111 + HX10-55	3 putative novel compounds related to actinoperylone	Polyketide	231, 325	[M + H] + 326.1597
		Polyketide	226, 275, 325	[M + H] + 543.1457
		Polyketide	223, 325	[M + H] + 617.1647
FXJ1.4112 + HX09-1	2 putative novel compounds	Unknown	200, 225, 278, 317, 331	[M + H] + 654.3387
		Unknown	196, 224	[M − H] − 717.4581
FXJ1.4112 + HX10-42	2 putative novel compounds related to collinomycin	Polyketide	220, 285, 413	[M − H] − 507.0928
		Polyketide	220, 285, 415	[M − H] − 521.1081
FXJ1.4112 + HX10-55	2 putative novel compounds	Unknown	227	[M + H] + 747.4677
		Unknown	197	[M + Na] + 691.5305
FXJ1.4122 + HX14-21	1 putative novel compound	Unknown	234, 278	[M + H] + 587.1566

## Data Availability

The data presented in this study are available in the article and supplementary material.

## Supplementary Materials

Supplementary data include figures, tables and associated description are available online at https://www.mdpi.com/article/10.3390/microorganisms9112187/s1, Table S1: Mycolic acid-containing bacteria (MACB) and streptomycetes used in this study. Table S2: Linear elution ratio of methanol and water in HPLC-PDA analysis. Table S3: HPLC-PDA/HRMS data of 1.264HX-**1**–**6**. Table S4: NMR data of 1.264HX-**3** in CDCl_3_ (500 MHz). Figure S1: The device for non-contact co-culture. Figure S2: HPLC and HRMS chromatograms of compounds 1.264HX-**1**–**6**. Figure S3: Three putative structures of 1.264HX-**3**. Figure S4: ^1^H spectrum of 1.264HX-**3**. Figure S5: ^13^C spectrum of 1.264HX-**3**. Figure S6: H-H COSY spectrum of 1.264HX-**3**. Figure S7: DEPT135 spectrum of 1.264HX-**3**. Figure S8: HMBC spectrum of 1.264HX-**3**. Figure S9: HSQC spectrum of 1.264HX-**3**. Figure S10: NOESY spectrum of 1.264HX-**3**. Figure S11: HPLC analysis of fermentation extracts of co-cultures of *S.* sp. FXJ1.264 and heat-killed *M.* sp. HX09-1. Figure S12: Comparison of activation effects of MACB on different actinobacteria. Figure S13: HPLC and HRMS analysis of fermentation extracts of *S*. sp. FXJ1.4012, *N*. sp. HX14-21, and their co-culture.
