# Supplementary material for: Activation of Secondary Metabolism in Red Soil-Derived Streptomycetes via Co-Culture with Mycolic Acid-Containing Bacteria
Source: Microorganisms. 2021 Oct 20;9(11):2187. doi: 10.3390/microorganisms9112187 (PMC8622677; doi:10.3390/microorganisms9112187)
Supplement: Supplementary file 1 [file microorganisms-09-02187-s001.zip › Coculture-supplementary-resubmit.pdf]

Supplementary Materials

Table S1 Mycolic acid-containing bacteria (MACB) and streptomycetes used in this study.

No.	Strain number	Genus	Most closely related type strain	16S rRNA gene similarity
1.	HX09-1	*Mycobacterium*	*Mycobacterium hodleri* JCM 12141^T	99.32 %
2.	HX09-8	*Rhodococcus*	*Rhodococcus qingshengii* djl-6^T	100.00 %
3.	HX09-20	*Nocardia*	*Nocardia cummidelens* R89^T	99.78 %
4.	HX10-42	*Mycobacterium*	*Mycobacterium madagascariense* P2^T	98.64 %
5.	HX10-55	*Rhodococcus*	*Rhodococcus opacus* DSM 43205^T	99.92 %
6.	HX11-30	*Rhodococcus*	*Rhodococcus yunnanensis* YIM 70056^T	99.70 %
7.	HX11-35	*Rhodococcus*	*Rhodococcus cercidiphylli* YIM 65003^T	99.77 %
8.	HX14-21	*Nocardia*	*Nocardia fluminea* DSM 44489^T	98.64 %
9.	WS03-62	*Nocardia*	*Nocardia abscessus* NBRC 100374^T	98.94 %
10.	WS04-9	*Rhodococcus*	*Rhodococcus qingshengii* JCM 15477^T	100.00 %
11.	FXJ1.050	*Streptomyces*	*Streptomyces hygroscopicus* subsp. CGMCC 4.1527^T	100.00 %
12.	FXJ1.068	*Streptomyces*	*Streptomyces hygroscopicus* subsp. CGMCC 4.1252^T	99.85 %
13.	FXJ1.069	*Streptomyces*	*Streptomyces yeochonensis* NBRC 100782^T	98.08 %
14.	FXJ1.076	*Streptomyces*	*Streptomyces hygroscopicus* subsp. NBRC 16551^T	100.00 %
15.	FXJ1.172	*Streptomyces*	*Streptomyces naganishii* NRRL B-1816^T	99.21 %
16.	FXJ1.235	*Streptomyces*	*Streptomyces lasalocidi* ATCC 31180^T	99.06 %
17.	FXJ1.253	*Streptomyces*	*Streptomyces hygroscopicus* subsp. NBRC 16551^T	100.00 %
18.	FXJ1.264	*Streptomyces*	*Streptomyces cuspidosporus* NBRC 12378^T	99.78 %
19.	FXJ1.4008	*Streptomyces*	*Streptomyces anandii* JCM 4720^T	99.24 %
20.	FXJ1.4012	*Streptomyces*	*Streptomyces diastaticus* subsp. *ardesiacus* NRRL B-1773^T	99.77 %
21.	FXJ1.4013	*Streptomyces*	*Streptomyces misionensis* NBRC 13063^T	100.00 %
22.	FXJ1.4014	*Streptomyces*	*Streptomyces naganishii* NBRC 12892^T	100.00 %
23.	FXJ1.4033	*Streptomyces*	*Streptomyces achromogenes* subsp. *rubradiris* NBRC 14000^T	99.25 %
24.	FXJ1.4034	*Streptomyces*	*Streptomyces aldersoniae* NRRL 18513^T	99.71 %
25.	FXJ1.4035	*Streptomyces*	*Streptomyces aurantiogriseus* NBRC 12842^T	98.43 %
26.	FXJ1.4037	*Streptomyces*	*Streptomyces avellaneus* NBRC 13451^T	99.63 %
27.	FXJ1.4038	*Streptomyces*	*Streptomyces chromofuscus* NBRC 12851^T	98.13 %
28.	FXJ1.4041	*Streptomyces*	*Streptomyces cuspidosporus* NBRC 12378^T	99.78 %
29.	FXJ1.4044	*Streptomyces*	*Streptomyces durhamensis* NRRL B-3309^T	98.83 %
30.	FXJ1.4054	*Streptomyces*	*Streptomyces rameus* LMG 20326^T	99.33 %
31.	FXJ1.4056	*Streptomyces*	*Streptomyces somaliensis* NBRC 12916^T	97.84 %
32.	FXJ1.4059	*Streptomyces*	*Streptomyces spiralis* NBRC 14215^T	100.00 %
33.	FXJ1.4061	*Streptomyces*	*Streptomyces thermoviolaceus* subsp. *apingens* DSM 41392^T	99.18 %
34.	FXJ1.4075	*Streptomyces*	*Streptomyces corchorusii* NBRC 13032^T	99.48 %
35.	FXJ1.4082	*Streptomyces*	*Streptomyces malachitofuscus* NBRC 13059^T	99.18 %

36.	FXJ1.4087	*Streptomyces*	*Streptomyces rameus* LMG 20326^T	99.26 %
37.	FXJ1.4088	*Streptomyces*	*Streptomyces spinoverrucosus* NBRC 14228^T	98.44 %
38.	FXJ1.4089	*Streptomyces*	*Streptomyces thermoviolaceus* subsp. *apingens* DSM 41392^T	99.18 %
39.	FXJ1.4097	*Streptomyces*	*Streptomyces azureus* NBRC 12744^T	99.93 %
40.	FXJ1.4098	*Streptomyces*	*Streptomyces bingchenggensis* BCW-1^T	99.70 %
41.	FXJ1.4099	*Streptomyces*	*Streptomyces caeruleatus* GIMN4^T	99.41 %
42.	FXJ1.4101	*Streptomyces*	*Streptomyces europaeiscabiei* KACC 20186^T	99.85 %
43.	FXJ1.4104	*Streptomyces*	*Streptomyces hygroscopicus* subsp. *ossamyceticus* NBRC 13983^T	99.93 %
44.	FXJ1.4106	*Streptomyces*	*Streptomyces cinereoruber* subsp. cinereoruber NBRC 12756^T	99.78 %
45.	FXJ1.4107	*Streptomyces*	*Streptomyces glauciniger* NBRC 100913^T	99.20 %
46.	FXJ1.4108	*Streptomyces*	*Streptomyces hygroscopicus* subsp. ossamyceticus NBRC 13983^T	100.00 %
47.	FXJ1.4109	*Streptomyces*	*Streptomyces panaciradicis* 1MR-8^T	99.26 %
48.	FXJ1.4110	*Streptomyces*	*Streptomyces pseudovenezuelae* NBRC 12904^T	99.18 %
49.	FXJ1.4111	*Streptomyces*	*Streptomyces thermoviolaceus* subsp. *apingens* DSM 41392^T	99.26 %
50.	FXJ1.4112	*Streptomyces*	*Streptomyces yaanensis* Z4^T	99.93 %
51.	FXJ1.532	*Streptomyces*	*Streptomyces ferralitis CGMCC*4.1985^T	99.19 %
52.	FXJ1.535	*Streptomyces*	*Streptomyces ferralitis* CGMCC4.1985^T	98.53 %
53.	FXJ1.907	*Streptomyces*	*Streptomyces spinoverrucosus* NBRC 14228^T	98.38 %
54.	FXJ23y	*Streptomyces*	*Streptomyces achromogenes* subsp. *rubradiris* NBRC 14000^T	99.19 %

Table S2 Linear elution ratio of methanol and water in HPLC-PDA analysis.

**Time (min)**	0	15	20.5	20.51	26.51
**ddH$_2$O (%)**	80	0	0	80	80
**Methanol (%)**	20	100	100	20	20

Table S3 HPLC-PDA/HRMS data of 1.264HX-**1**–**6**.

**Chemical data**	1.264HX-**1**	1.264HX-**2**	1.264HX-**3**	1.264HX-**4**	1.264HX-**5**	1.264HX-**6**
HRMS (positive)	$[M+H]^+$ 373.1432	–	$[M+H]^+$ 387.1599	–	–	–
HRMS (negative)	$[M-H]^-$ 371.1292	–	$[M-H]^-$ 385.1433	$[M-H]^-$ 743.2671	$[M-H]^-$ 743.2645	$[M-H]^-$ 743.2634
Molecular formula	$C_{24}H_{20}O_4$	–	$C_{25}H_{22}O_4$	$C_{48}H_{40}O_8$	$C_{48}H_{40}O_8$	$C_{48}H_{40}O_8$
Molecular weight	372	–	386	744	744	744
UV absorptivity (nm)	195, 251, 285, 376	195, 251, 285, 376	195, 251, 285, 376	195, 251, 285, 376	195, 251, 285, 376	195, 251, 285, 376

Note: '–', no corresponding signal detected in mass spectrum.

Table S4 NMR data of 1.264HX-**3** in *CDCl₃* (500 MHz).

Number	Chemical shift of ^{13}C (ppm)	Chemical shift of ^{1}H (ppm, J in Hz)	DEPT135
1	13.3	1.53 (d)	CH_3
2	28.7	1.18 (s)	CH_2
3	32.49	3.6 (d)	CH_2
3-1?	32.51	3.6 (d)	CH_2
4	54.7	3.8 (s)	CH_3
5	85.72	5.00 (m)	CH
5-1?	85.69	5.00 (m)	CH
6	88.54	5.12 (m)	CH
6-1?	88.57	5.12 (m)	CH
A	99.14		C
A-1?	98.79		C
7	99.69	6.40 (d, 2.5)	CH
7-1?	99.99	6.415 (d, 2.5)	CH
8	101.01	6.30 (d, 2.5)	CH
8-1?	101.0	6.307 (d, 2.)	CH
9	105.22	6.24 (s)	CH
9-1?	106.71	6.29 (s)	CH
10	121.23	6.15 (d, 15.5)	CH
10-1?	117.49	5.80（d, 12）	CH
11	124.4	7.5 (d, 4)	CH
11-1?	125.08	7.5 (d, 4)	CH
12	125.67	7.14 (m)	CH
12-1?	125.75	7.14 (m)	CH
13	125.99	7.14 (m)	CH
14	127.46	6.78，6.75 (dd, 11.2, 15.5)	CH
14-1?	127.39	7.91，7.88 (dd, 11.5, 15.5)	CH
15	128.98	7.14 (m)	CH
15-1?	128.81	7.14 (m)	CH
16	133.38	7.2 (d, 11)	CH
17	133.67	7.04 (d, 15.5)	CH
18	133.95	6.97 (d, 15.5)	CH
19	134.35		C
19-1?	134.38		C
19-2?	135.13		C
20	137.58		C
20-1?	137.41		C
21	138.29		C
21-1?	138.19		C
22	151.4		C
22-1?	152.9		C
23	162.75		C
23-1?	162.65		C
24	164.58		C
24-1?	164.43		C

25	165.73	C
25-1?	165.68	C
26	204.23	C=O
26-1?	204.20	C=O

Note: '?', the chemical shifts of ^{13}C have not yet been determined.

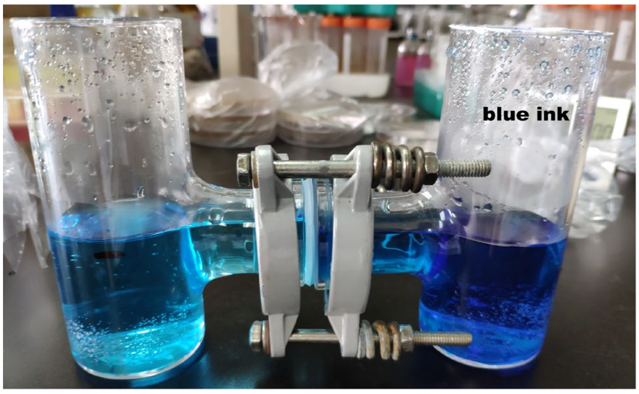

Figure S1 The device for non-contact co-culture. Diffusion of blue ink through a 0.22-μm polyether sulfone membrane from the right compartment to the left was observed within 2 hours.

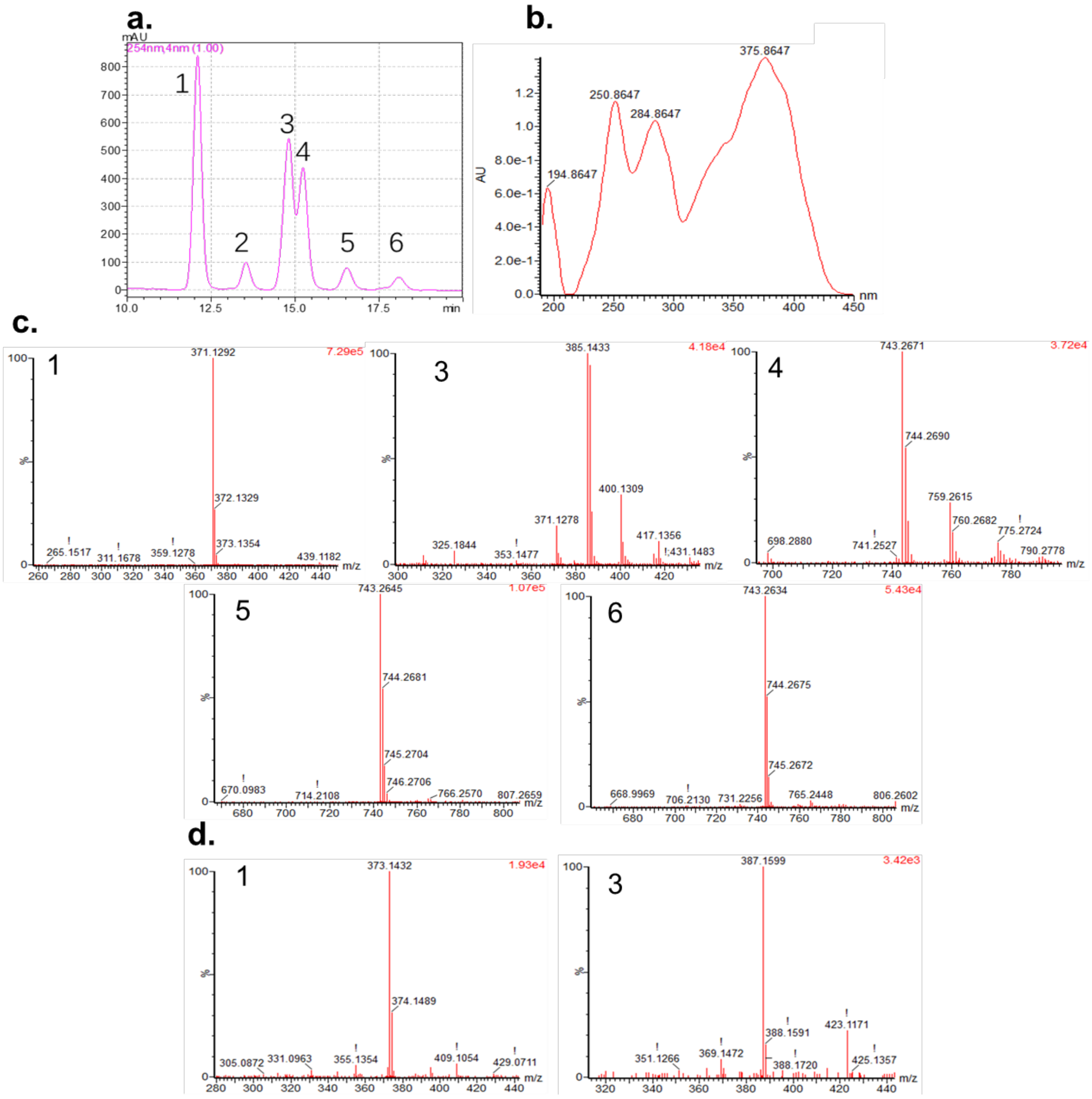

Figure S2 HPLC and HRMS chromatograms of compounds 1.264HX-**1**–**6**; (**a**) HPLC chromatogram of 1.264HX-**1**–**6**; (**b**) Ultraviolet absorption data of 1.264 HX-**1**–**6** at 254 nm; (**c**) HRMS data (negative ion mode) of 1.264 HX-**1**–**6** corresponding to the absorption peaks in (**a**); (**d**) HRMS data (postive ion mode) of 1.264 HX-**1** and 1.264 HX-**3**.

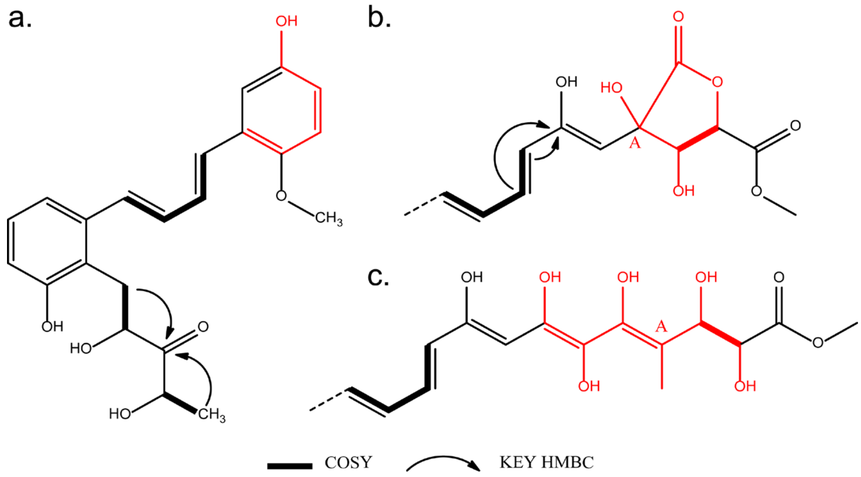

Figure S3 Three putative structures of 1.264HX-**3**; the black and red parts in the diagram represents the determined and undetermined structure elucidation, respectively.

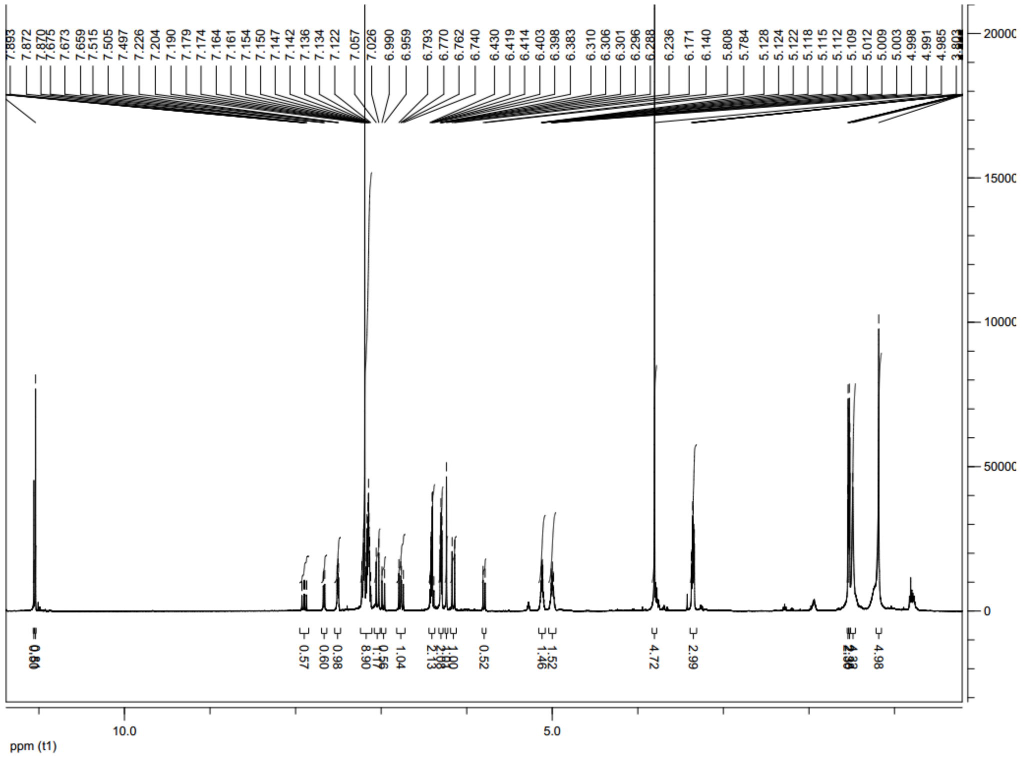

Figure S4 1H spectrum of 1.264HX-**3**.

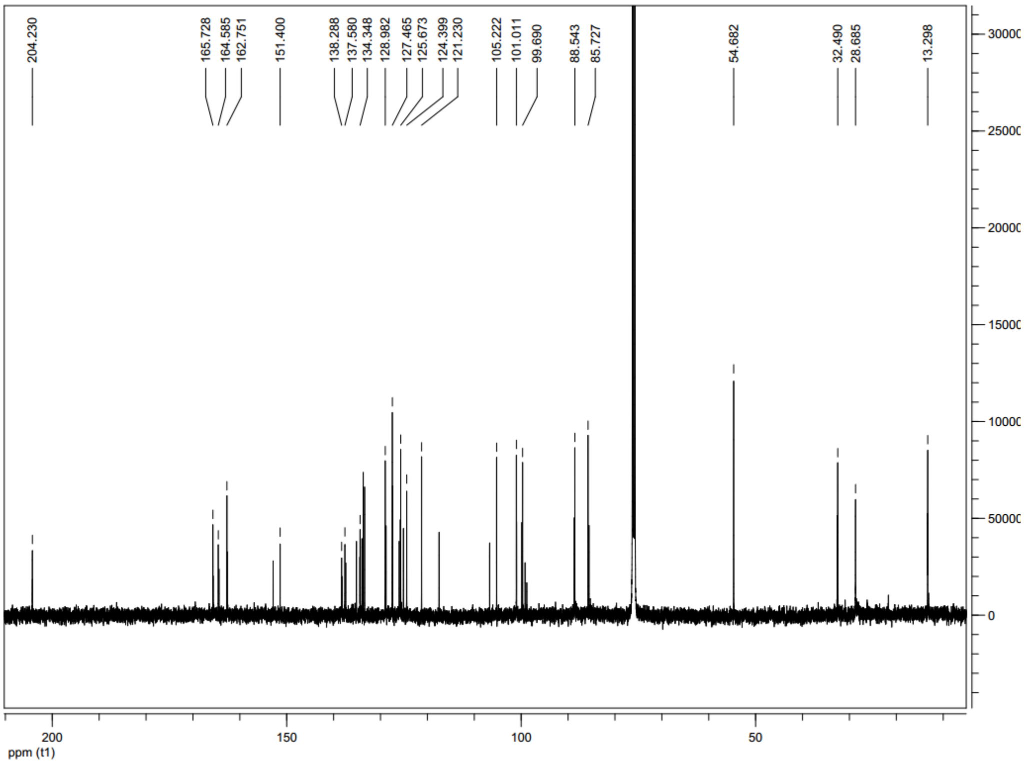

Figure S5 ^{13}C spectrum of 1.264HX-**3**.

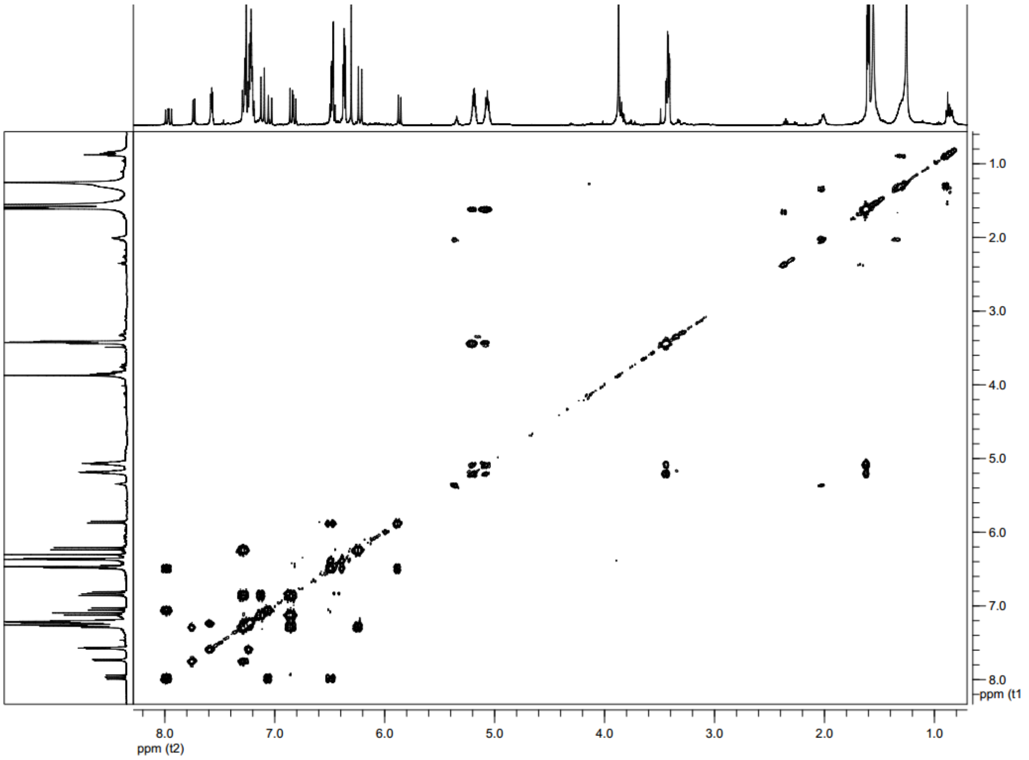

Figure S6 H-H COSY spectrum of 1.264HX-**3**.

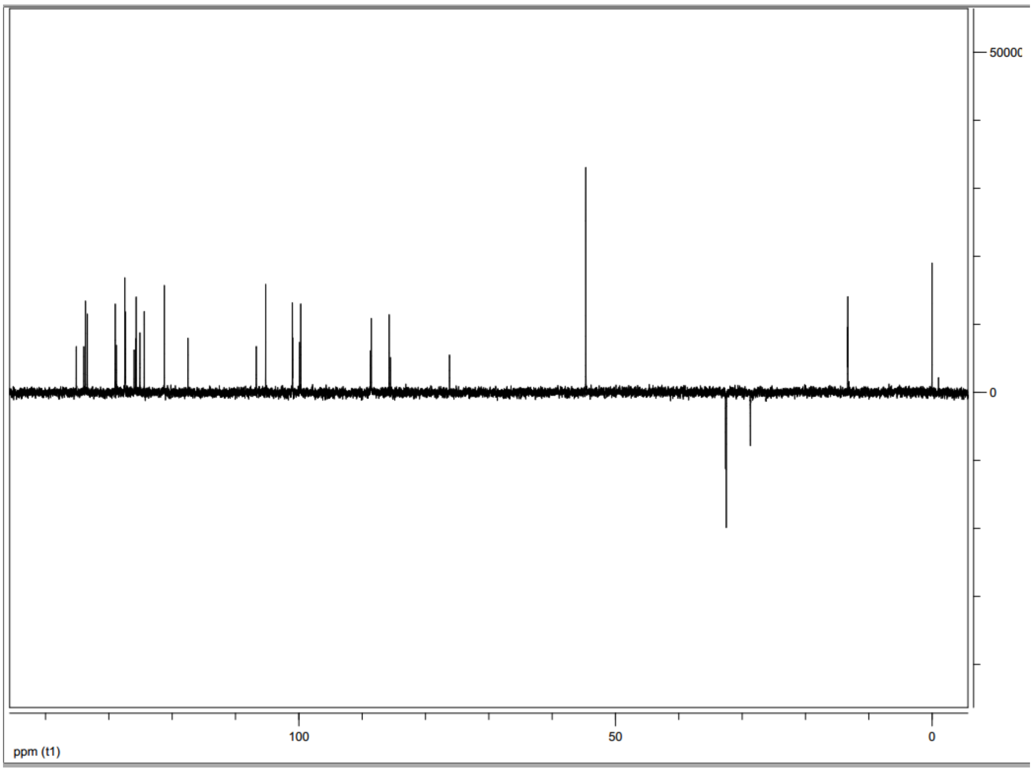

Figure S7 DEPT135 spectrum of 1.264HX-**3**.

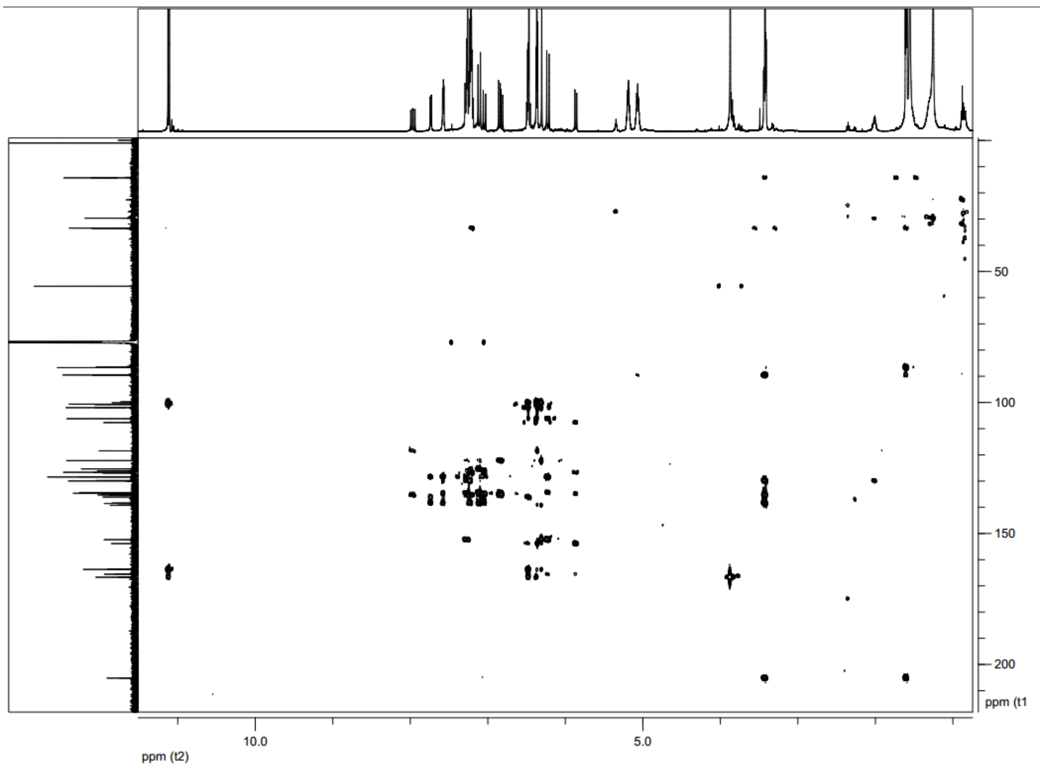

Figure S8 HMBC spectrum of 1.264HX-**3**.

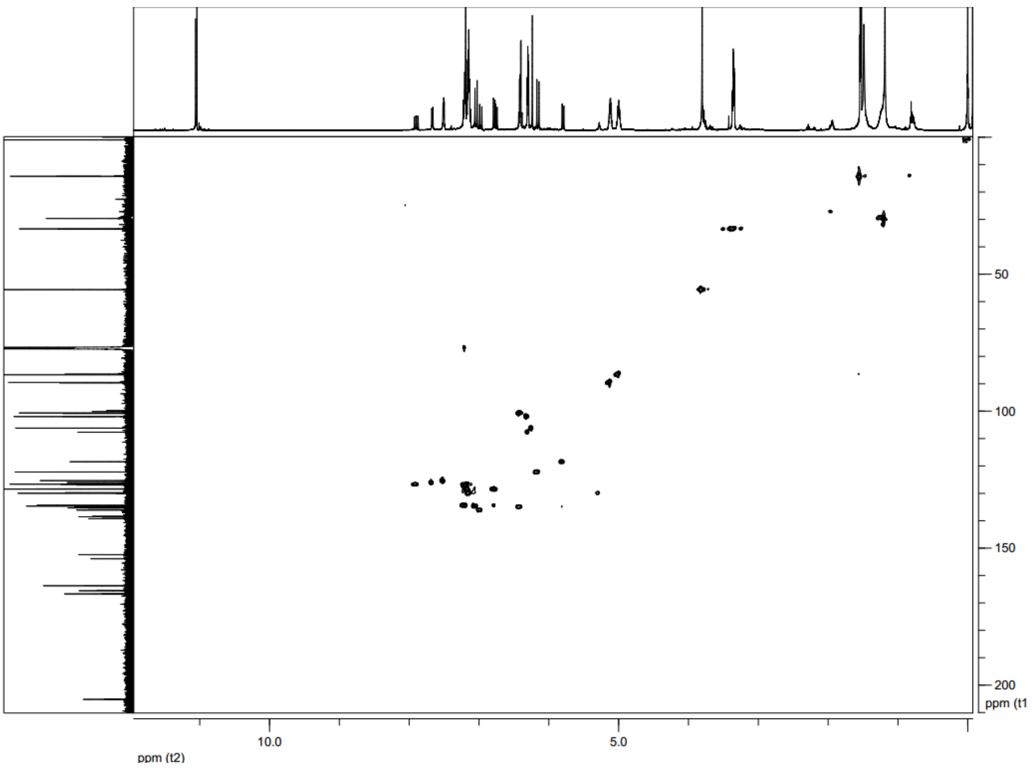

Figure S9 HSQC spectrum of 1.264HX-**3**.

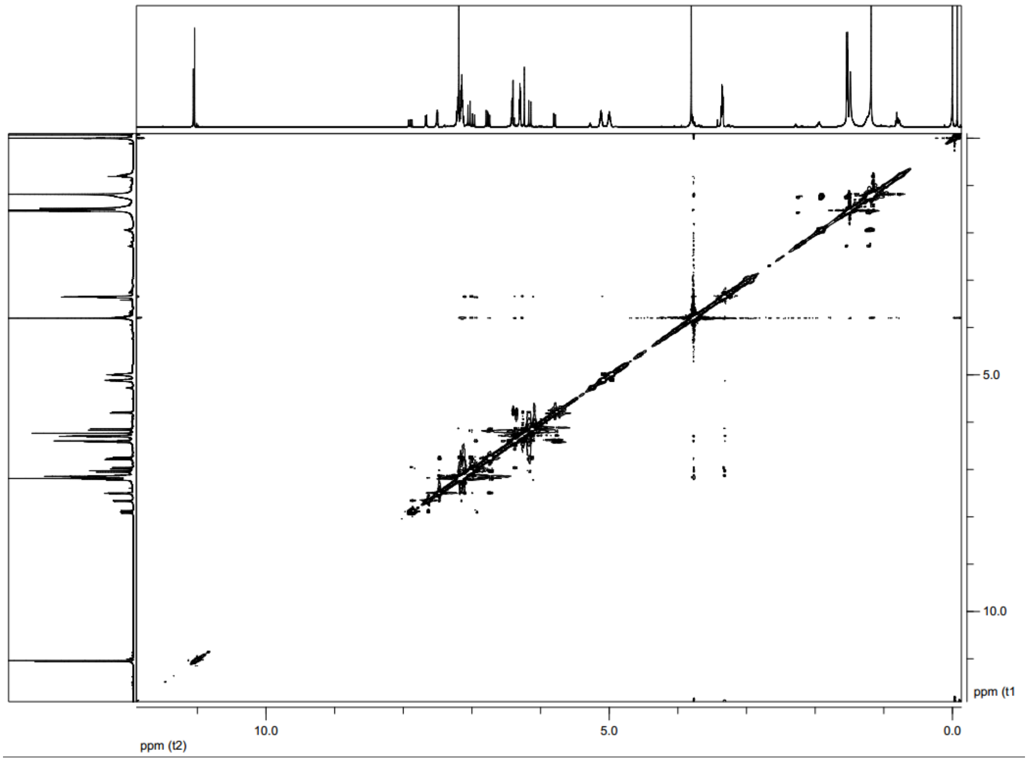

Figure S10 NOESYspectrum of 1.264HX-**3**.

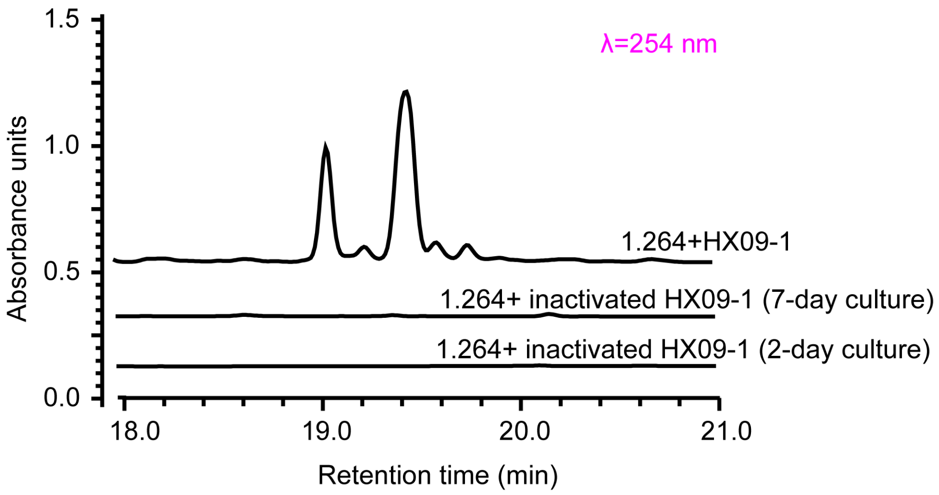

Figure S11 HPLC analysis of fermentation extracts of co-cultures of *S*. sp. FXJ1.264 and heat-killed *M*. sp. HX09-1.

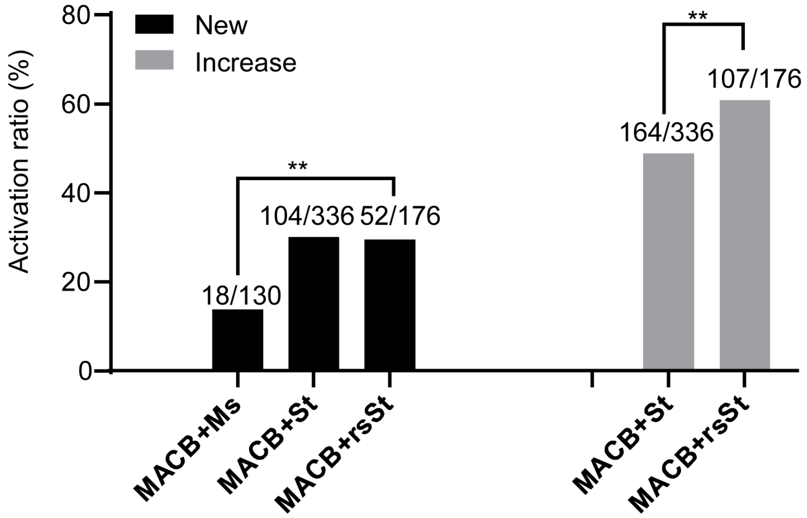

Figure S12 Comparison of activation effects of MACB on different actinobacteria.

Ms, marine *Micromonosporaceae*; St, *Streptomyces*; rsSt, red soil-derived *Streptomyces*; New, 'new' products detected in co-cultures; Increase, the increase in the yield of original metabolites; MACB+Ms, co-cultures of MACB and *Micromonosporaceae* [1]; MACB+St, co-cultures of MACB and *Streptomyces* [2]; MACB+ rsSt, co-cultures of MACB and red soil-derived *Streptomyces* (this study); **, p<0.01; the increase in the yield of original metabolites of MACB+Ms is unavaliable.

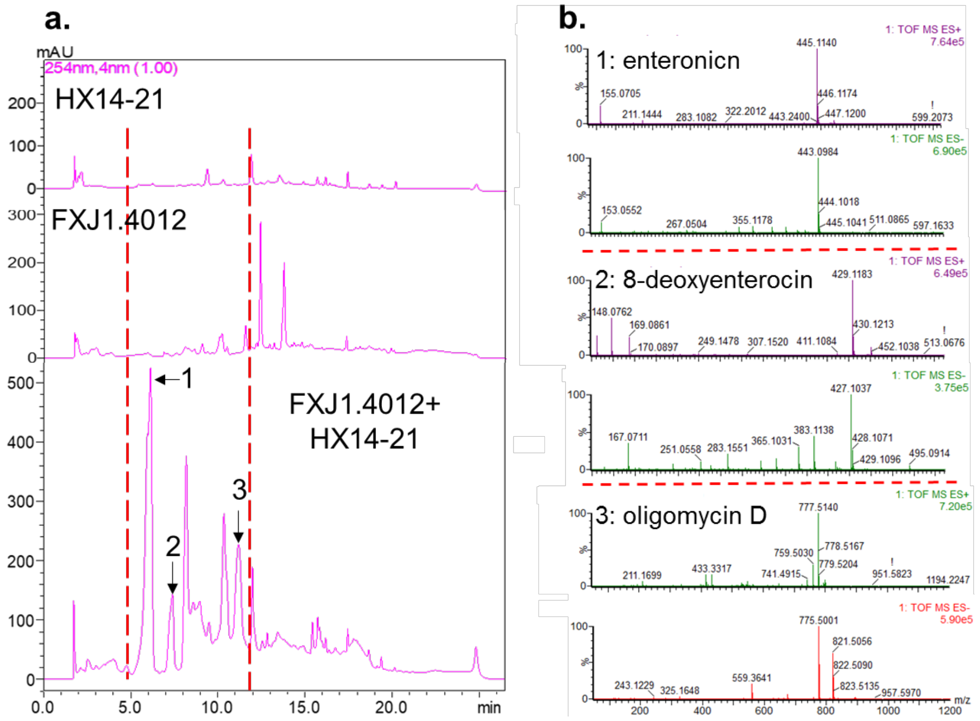

Figure S13 HPLC and HRMS analysis of fermentation extracts of *S*. sp. FXJ1.4012, *N*. sp. HX14-21, and their co-culture. (**a**) HPLC analysis of fermentation extracts of *S*. sp. FXJ1.4012, *N*. sp. HX14-21, and their co-culture; (**b**) HRMS data corresponding to peaks in **a**. $[M+H]^+$=445.1140 and $[M+H]^-$=443.0984 for enterocin; $[M+H]^+$=429.1183 and $[M+H]^-$=427.1037 for 8-deoxyenterocin; $[M+H]^+$=777.5140 and $[M+H]^-$=775.5001 for oligomycin D.

References

1. Adnani, N.; Vazquez-Rivera, E.; Adibhatla, S.N.; Ellis, G.A.; Braun, D.R.; Bugni, T.S. Investigation of interspecies interactions within marine *Micromonosporaceae* using an improved co-culture approach. *Mar. Drugs* **2015**, *13*, 6082-6098.
2. Onaka, H.; Mori, Y.; Igarashi, Y.; Furumai, T. Mycolic acid-containing bacteria induce natural-product biosynthesis in *Streptomyces* species. *Appl*. *Environ*. *Microbiol*. **2011**, *77*, 400-406.
